# Role of Complement Receptors (CRs) on DCs in Anti-HIV-1 Immunity

**DOI:** 10.3389/fimmu.2020.572114

**Published:** 2020-11-03

**Authors:** Wilfried Posch, Marta Bermejo-Jambrina, Cornelia Lass-Flörl, Doris Wilflingseder

**Affiliations:** ^1^ Institute of Hygiene and Medical Microbiology, Medical University of Innsbruck, Innsbruck, Austria; ^2^ Department of Experimental Immunology, Amsterdam Infection and Immunity Institute, Academic Medical Center, University of Amsterdam, Amsterdam, Netherlands

**Keywords:** complement receptors, dendritic cells, HIV-1, antiviral immunity, immune activation, SAMHD1, antigen presentation, type I IFN

## Abstract

Upon entry of human immunodeficiency virus 1 (HIV-1) into the host, innate immune mechanisms are acting as a first line of defense, that considerably also modify adaptive immunity by the provision of specific signals. Innate and adaptive immune responses are intimately linked and dendritic cells (DCs) together with complement (C) play an important role in regulation of adaptive immunity. Initially, the role of complement was considered to primarily support – or COMPLEMENT - cytolytic actions of antibodies or antibody-complexed antigens (immune complexes, ICs) or directly kill the pathogens by complement-mediated lysis. Recently, the role of complement was revised and found to significantly augmenting and modulating adaptive immunity, in particular against viruses. Complement and DCs are therefore predestined to open novel avenues for antiviral research and potential therapeutic interventions. Recent studies on interactions of complement-opsonized HIV-1 with DCs demonstrated a high potential of such primed DCs to initiate efficient antiviral and cytotoxic anti-HIV-1 immunity and complement-coated viral particles shift DCs functions *via* CR3 and CR4 in an antithetic manner. This review will focus on our current knowledge of CR3 and CR4 actions on DCs during HIV-1 binding and the outcome of infection influenced by entry and signaling pathways.

## Introduction

Innate immunity is the first line of defense against pathogens. It acts in a non-specific manner but is rapid and independent of antigen. The innate immune system is formed by cellular and humoral components. Complement comprises a part of the humoral innate immune system. The complement system recognizes, opsonizes and subsequently lyses the pathogens and additionally also induces pro-inflammatory cytokines and maintains homeostasis. Immediately upon Human Immunodeficiency Virus 1 (HIV-1) entry in the body, the virus spontaneously activates the complement system even in the absence of specific antibodies. This is possible due to the complement component C1q binding site in the envelope glycoprotein gp41 of HIV-1 (1). Thus, HIV-1 is already coated with complement fragments at the initial stages of infection and during transfer. Following seroconversion, in addition to complement fragments, specific antibodies coat the viral surface. Dependent on the IgG subtype, formation of immune-complexes strongly enhances the activation of the complement system *via* the classical pathway and virus-bound antibodies dramatically increase the deposition of complement fragments (C3b) on virions (2–4). Therefore, opsonized infectious viral particles accumulate in HIV-1-positive individuals during the acute and chronic phases of infection.

Most HIV-1 particles are not killed by complement-mediated lysis but persist covered with C3 fragments in the host. This is due to the uptake of regulators of the complement activation (RCA) by the viral particles during the budding process. RCA tightly control the complement system to prevent spontaneous destruction of host cells and unfortunately they also protect HIV-1 from being lysed (5). Interestingly, opsonized HIV-1 accumulates in all so far tested compartments of HIV-1-positive individuals, for instance mucosa or seminal fluid (6). As such, it is able to interact with complement receptor (CR)-expressing cells, like dendritic cells (DCs) or macrophages. Triggering these receptors leads to cell activation and contributes to inflammation.

On one hand, complement-opsonized HIV-1 (HIV-C) increases viral infectivity and transmission *in vitro* (7). On the other hand, it strengthens cellular immunity as well as type I IFN responses (8–10). This highlights the importance of complement-mediated processes during HIV-1 pathogenesis.

One of the most important cellular components of the innate immune system are dendritic cells (DCs). They play a major role in induction of immune responses against pathogens, allergies and cancer (11, 12). Interestingly, they differ in their ability to induce an innate immune response against non-opsonized HIV-1, complement-opsonized HIV-1 and HIV type 2 (HIV-2) (9, 13, 14). Both HIV-1 and HIV-2 are causing an immunodeficiency syndrome, but differ in their genome, tropism, infectivity and pathogenicity (15). One of the most important differences is the reduced activation and infection of DCs by HIV-1, that was described to inefficiently infecting and activating DCs whereas HIV-2, and surprisingly also HIV-C, are able to efficiently infect and activate DCs (9, 10, 13, 16).

**Figure 1:**
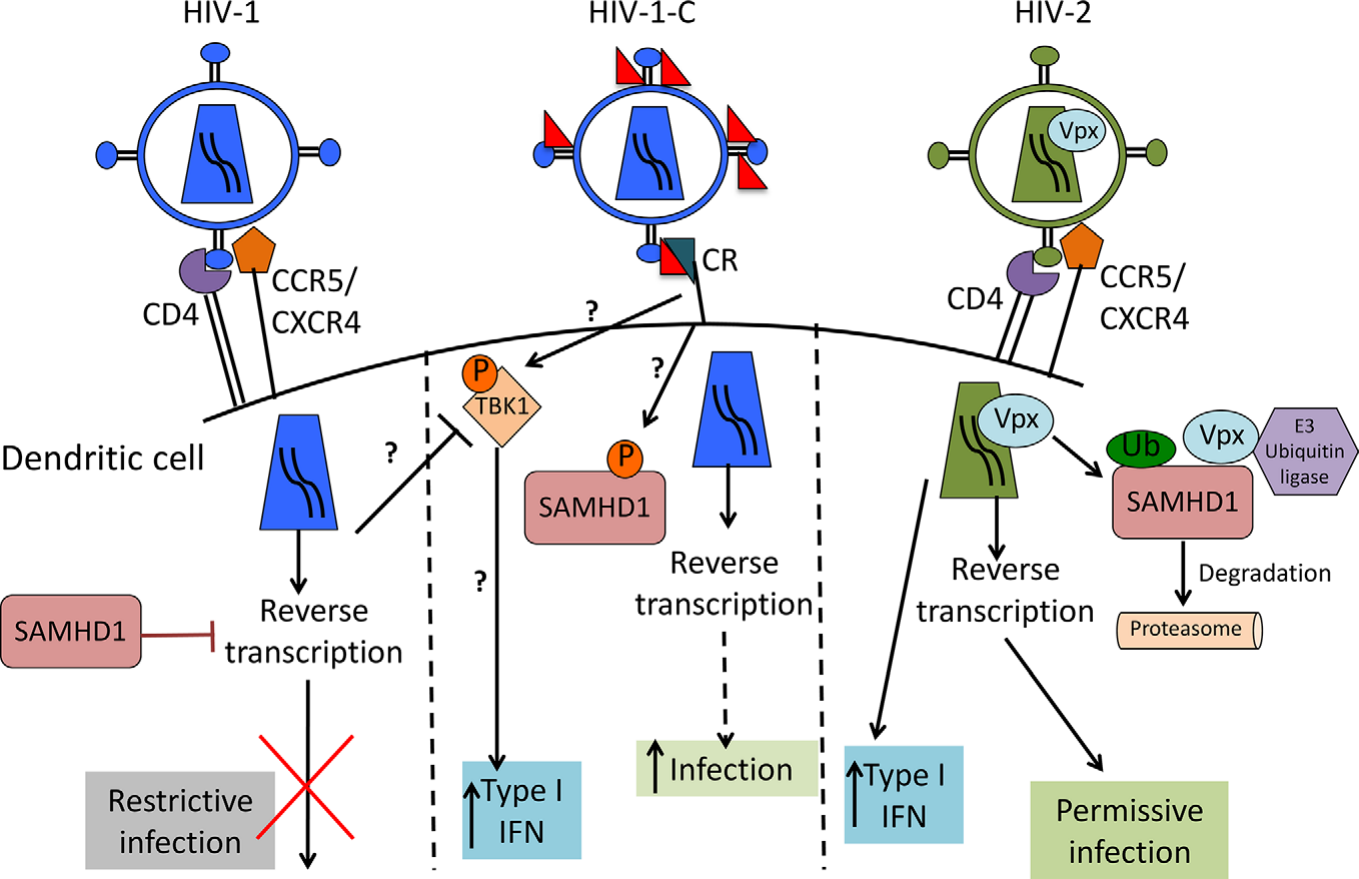
Scheme of HIV-1, complement-opsonized HIV-1 (HIV-C) and HIV-2 restriction by SAMHD1 in DCs. HIV-1 (left) binds to CD4 and CCR5 or CXCR4 to enter the cell. SAMHD1 action inhibits HIV-1 reverse transcription and blocks infection. HIV-C (middle) binds to complement receptors (CRs), which trigger a yet unknown pathway that leads to SAMHD1 inactivation by phosphorylation. This pathway makes the virus visible for DCs by permitting reverse transcription and significantly higher DC infection as well as increased type 1 IFN induction than with HIV-1. HIV-2 (right) contains Vpx that mediates SAMHD1 degradation, thereby allowing reverse transcription to occur, productive infection and higher type 1 IFN as also seen with HIV-C.

**Figure 2:**
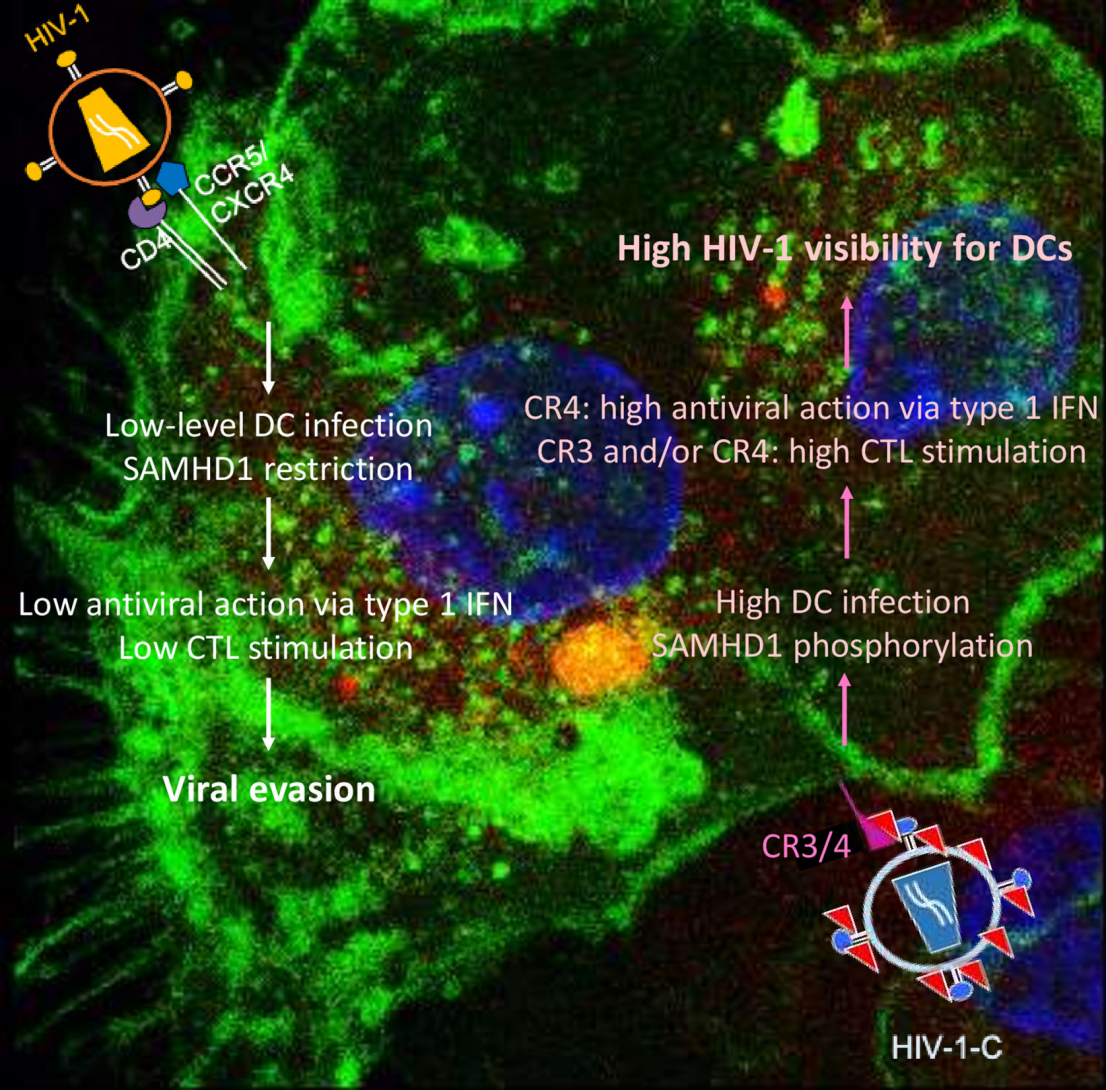
Summary of DC functions modulated by non-opsonized HIV-1 (left) and complement-opsonized HIV-1 (HIV-C, right). Non-opsonized HIV-1 only causes low-level productive DC infection due to SAMHD1 restriction, which is associated with weak antiviral induction (left). In contrast, HIV-C overcomes SAMHD1 restriction in DCs by T592 phosphorylation—this is connected to induction of efficient type 1 IFN and CTL responses *via* DCs.

Differences between HIV-1 and HIV-2 are mainly caused by viral protein x (Vpx), the HIV-2 accessory protein that leads to SAMHD1 degradation (17–19), probably one of the most important HIV-1 restriction factors in myeloid cells. SAMHD1 is a deoxynucleoside-triphosphate (dNTP) triphosphohydrolase that restricts the replication of HIV-1 in non-cycling monocytes, monocyte-derived macrophages (MDMs), DCs, and resting T-cells (17, 19, 20). It depletes the intracellular pool of dNTPs, resulting in a blockade of virus replication at the step of reverse transcription (19, 20). Since HIV-1 lacks Vpx, it has no means to counteract the restriction. The antiviral activity of SAMHD1 is regulated by phosphorylation of amino acid T592, which results in the loss of antiviral restriction activity (21, 22). In cycling T cells, SAMHD1 is constitutively phosphorylated by cyclin dependent kinase 1 (CDK1) and does not restrict HIV-1 replication (23). In myeloid and resting lymphoid cells, where SAMHD1 exists as a mixture of phosphorylated and dephosphorylated forms, the phosphorylation is mediated by CDK2 (24). Restriction factor expression and their regulation are among the most important factors dictating HIV-1 infection of a specific cell. Specifically, after different stimuli, phosphorylation of SAMHD1 modifies its ability, leading to the inhibition of HIV-1 infection in macrophages (25, 26). Importantly, complement-opsonized HIV-1 negatively regulates SAMHD1 in DCs by inducing its phosphorylation, which results in significantly higher DC infection with HIV-C compared to non-opsonized HIV-1, even though there is no Vpx (9) (**Figure 1**). These facts illustrate that HIV-C behaves more similar in DCs to HIV-2 since the infection leads to a higher DC maturation and type I IFN expression (9). In contrast, non-opsonized HIV-1 only causes low-level productive infection of DCs due to SAMHD1 restriction and this low-level infection is associated with low-level antiviral actions and CTL induction, thus viral evasion (**Figure 2**).

These different features with respect to HIV-1 and complement-opsonized HIV-1 and previously described interactions with either complement receptor 3 (CR3, CD11b/CD18) or complement receptor 4 (CR4, CD11c/CD18) expressed on DCs is summarized below.

## DC Functions in HIV-1 Infection

DCs are localized in the skin and mucosa, including oral and vaginal mucosal surfaces, and in lymphoid tissues. Because of their location through all peripheral tissues and their high abundance of pattern recognition receptors (PRRs), DCs are among the first cells to encounter a plethora of pathogens and they are also called sentinels of the immune system (12, 27). DCs capture and internalize invading pathogens and process antigens on major histocompatibility complex (MHC) class I and II to present them to CD8^+^ and CD4^+^ T cells, respectively (28–30). DCs are key regulators of the immune system since they contribute to a broad spectrum of immune responses against infectious microbes, allergies and cancer (12, 28, 29, 31). A major function of DCs is the induction of an efficient adaptive immune response in lymph nodes (LNs); yet, DCs additionally interact with innate immune cells such as NK cells and NKT cells (32, 33). As immature DCs (iDCs), they reside in peripheral tissues, which they survey for invading pathogens. DCs express numerous PRRs that interact with PAMPs including the C-type lectin receptors (CLRs) [rev. in (34)] and the complement receptors (CRs). These PRRs not only interact with pathogens but also trigger signaling cascades (34). Upon pathogen encounter, DCs undergo maturation expressing molecules on their surface, which are important for antigen-presentation and T cell stimulation, such as CD40, CD80, CD83, CD86, and HLA. Further, they start migrating to the draining LNs *via* a chemokine gradient in a CCR7-dependent fashion (35, 36). In secondary lymphoid organs, they present the antigen to naïve CD8^+^ and CD4^+^ T cells, being the only antigen-presenting cells capable of activating naïve T cells (12, 29). Following the recognition of the peptide MHC, antigen-specific CD8^+^ and CD4^+^ T cells clonally expand and develop effector functions, such as cytokine production and cytotoxic activity. Effector T cells are recruited to the inflamed peripheral tissues and participate in the elimination of pathogens and infected cells. This very particular life cycle emphasizes to call DCs ‘sentinels of the immune system’ and this is why they are considered the bridge between the innate and adaptive immune system. When DCs get infected, they are able to sense HIV-1 and induce an innate immune response. One of the triggers for the innate immune activation is the recognition of nucleic acids by diverse sensors in the cytoplasm. HIV-1 DNA has been shown to be recognized by cGAS, that leads to type I IFN expression through STING activation (37). However, type I IFN expression in DCs upon HIV-1 infection is controversially discussed in the field, since many groups only find background levels of this cytokine (38). Interestingly, HIV-C or HIV-2 infection triggers a significantly higher DC maturation than non-opsonized HIV-1 (9, 13). However, the viral pathogen-associated molecular pattern (PAMP) for the higher visibility of complement-opsonized HIV-1 or HIV-2 is not known in DCs yet (9, 13, 17). The natural balance between DC infection and innate sensing in HIV-2 might contribute to the reduced pathogenicity of this virus, whereas the ability of HIV-1 to prevent sensing of its cDNA might contribute to its immune escape. Contrary to what happens during infection of DCs with non-opsonized HIV-1, complement-opsonized HIV-1 or HIV-2 strongly activate DCs, which results in activation of specific CD4^+^ and CD8^+^ T cells, starting an adaptive immune response.

## The Role of DCs During HIV-1 Infection

By clustering and activating T cells, DCs may activate antiviral immunity as well as facilitate the spread of the virus. *In vitro* experiments indicate that DCs efficiently capture and transmit HIV-1 to T cells and initiate a vigorous infection (31, 39–41). This implies that HIV-1 exploits DCs at mucosal sites to be transported to CD4^+^ T cells in the LNs *in vivo*. Virus is very efficiently transmitted from DCs to T cells either without or *via de novo* infection (41). In particular C-type lectins, such as DC-specific ICAM-3-grabbing non-integrin (DC-SIGN), expressed on DCs is implicated in the transfer of HIV-1 to T cells in the LNs (31, 42–45) [rev. in (34)]. In case of opsonized HIV, the DC-SIGN-dependent interactions play a minor role for the attachment of HIV-1 to DCs as well as the DC-mediated HIV-infection (46). However, complement-opsonization of HIV-1 significantly enhanced the productive infection of DCs compared to non-opsonized HIV-1 and additionally acted as an endogenous adjuvant for the DC-mediated induction of virus-specific cytotoxic T cells (CTLs) (8, 9). These results emphasize a role of different DC subtypes in the induction of an adaptive T cell response to complement-coated HIV-1. Indeed, a previous study revealed that complement-opsonization of HIV-1 prior to DC loading significantly enhanced the CD8^+^ T cell-stimulatory capacity and contributed to the induction of CD4^+^ effector function namely secretion of interleukin 17 (IL-17) (10). Overall, there is compelling evidence for the crucial role of the complement system and different subtypes of DCs in the polarized induction T cell immune responses in fighting HIV-1 and known interactions are summarized below. Nevertheless, there are still many gaps of knowledge with respect to DC processing of complement-opsonized HIV-1, which could improve current HIV-1 vaccination strategies.

## Complement Activation Upon HIV-1 Infection

**Figure 3:**
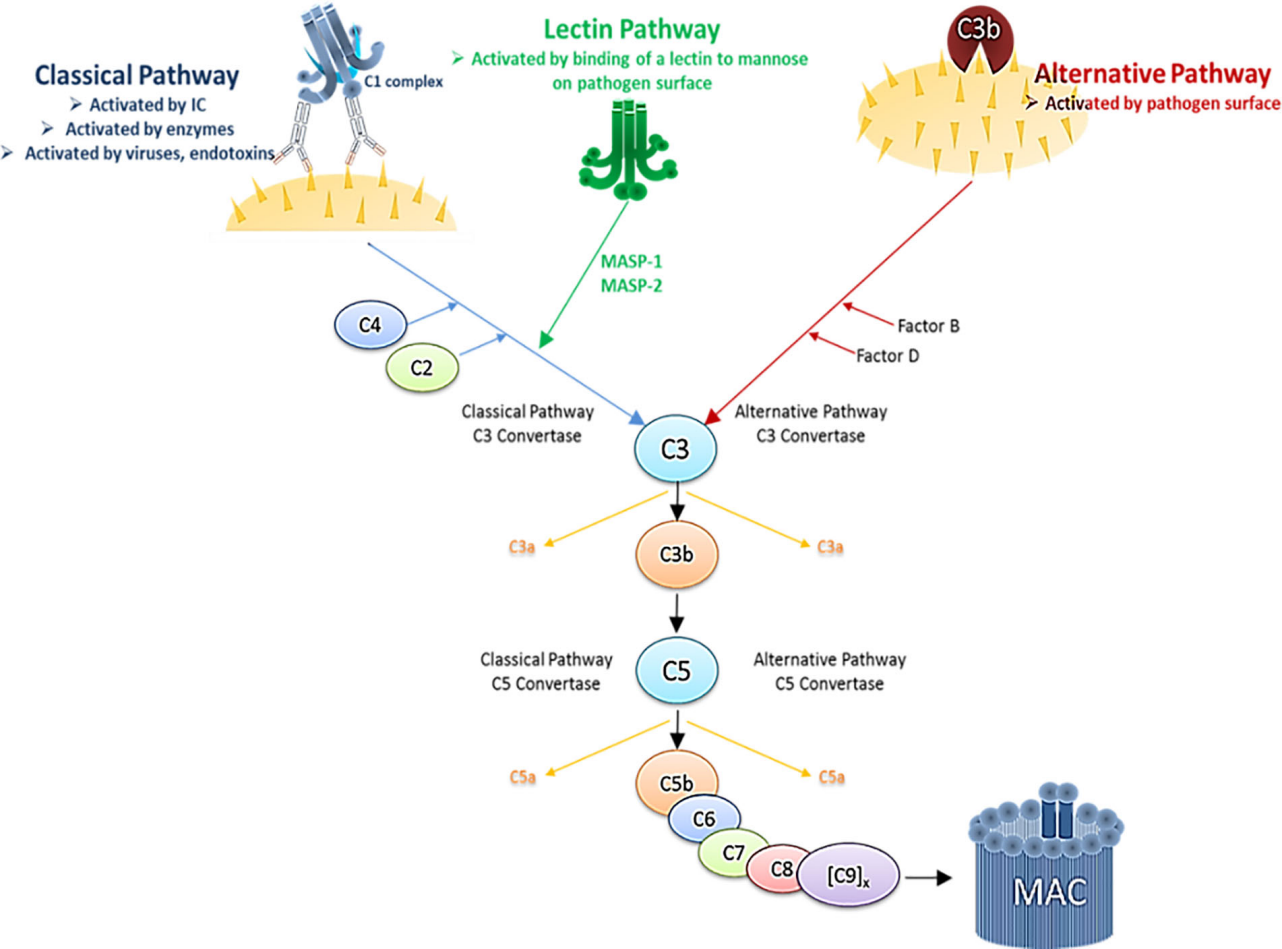
Simplified schematic illustration of the complement cascade depicting the three activation pathways. Immune complex (IC), Mannan-binding lectin serine protease 1/2 (MASP1/2).

The immune system comprises complex cellular and humoral responses, which form an interactive network to recognize and eradicate invading pathogens. Among the first humoral components activated during the innate immune response is the systemic complement (C) system. It consists of various fluid-phase and cell membrane-bound proteins and acts as a tightly regulated cascade of enzymatic reactions to defend the host against pathogens. Thus, the complement system represents a first line of host defense by opsonizing and subsequently lysing pathogens, inducing pro-inflammatory cytokines and maintaining homeostasis. It can be activated *via* three different biochemical pathways: the classical (CP), the alternative (AP) and the lectin pathway (LP) (**Figure 3**). Activation *via* the CP occurs when the first component, C1, binds the Fc region of either natural or antigen-specific immunoglobulin G (IgG) antibody immune-complexed with its antigen (Ag). The classical pathway can also be triggered in an antibody-independent manner when C1q directly binds to pathogens or infected cells, as it is the case with HIV-1 that has a C1q-binding side in its envelope glycoprotein (47–49). The AP is activated during normal physiological conditions at very low levels and monitors for certain microbial structures. Besides direct activation the alternative pathway can also be activated spontaneously by a process known as tick-over and thereby boost the C activation initiated by the classical or the lectin pathway. The LP is activated upon recognition of terminal mannose residues on microbial glycoproteins and glycolipids by mannose-binding lectin (MBL). All three pathways converge in the cleavage of the main C component 3 (C3) into the anaphylatoxin C component 3a (C3a) and the opsonin C component 3b (C3b). This cleavage initiates a cascade of further activation events. C3b is covalently deposited on microbial surfaces and together with the C3 convertase forms the C5 convertase, which then cleaves C component 5 (C5) into the anaphylatoxin C component 5a (C5a) and the C component 5b (C5b). C5b triggers the formation of the membrane attack complex (MAC), that consists of C5b, C component 6 (C6), C component 7 (C7), C component 8 (C8) and polymeric C component 9 (C9) molecules. The formation of the MAC disrupts the microbial membrane resulting in lysis of the pathogens or infected cells (**Figure 3**).

The C system is tightly controlled by RCAs to prevent spontaneous activation or destruction of bystander cells. RCAs are present in fluid-phase and also membrane-bound to protect host cells. The anaphylatoxins C3a and C5a are small fragments, which result from the cleavage of C3 and C5 respectively—C3a and C5a promote migration of immune cells to sites of infection and thus have a critical role in the activation of immune cells. Especially complement receptors (CRs), such CR3 and CR4 expressed on dendritic cells and macrophages, are responsible for detection of C3-opsonized microbes followed by activation of the immune cells and uptake of the pathogen. Furthermore, myeloid cells express C3a and C5a receptors (C3aR and C5aR, respectively) and triggering these receptors results in cell activation and contribution to inflammation by the complement system. Since C3aR and C5aR are also expressed by non-myeloid cells like B and T lymphocytes the complement system also influences the adaptive immunity. Crosstalk of C3aR and C5aR signaling with other intra- or extracellular receptor signaling is very likely, because of mutual interaction partners such as nuclear factor kappa-light-chain-enhancer of activated B cells (NF-kB) and Mitogen-activated protein kinases (MAPKs).

C3 opsonization of pathogens is important in the induction and maintenance of B cells. Additionally, opsonized immune complexes bind to CR2 on follicular dendritic cells in germinal centers to present antigens, which results in induction of effector and memory B cells or also trapping of intact virus particles within germinal centers, i.e. HIV-1 (50).

Besides the liver-derived systemic complement, immune cell-derived complement or cellular complement has been recently described to play a significant role in shaping immune responses (51–55). This local complement can be produced by almost all immune cells at sites of infection and is secreted by or stored inside the immune cells. Intracellular C, especially C3, is cleaved permanently by proteases such as cathepsins and binds to intracellular C3aRs on lysosomes. This engulfment regulates cell growth, cell proliferation and cell survival *via* the serine/threonine protein kinase mammalian target of rapamycin (mTOR) (56). Experiments co-culturing antigen-presenting cells (APCs) and T cells furthermore demonstrated the importance of cellular complement in terms of T helper cell development and proliferation. Interestingly, absence of cellular complement signals in these studies could not be compensated by systemic complement (52). Involvement of intra- and extracellular C has been investigated in T lymphocytes in more detail but only few studies investigating local production of C in DCs and macrophages were conducted. These studies reported that similar to T lymphocytes, DCs are also activated *via* anaphylatoxin receptors in an autocrine manner but it remains unknown which signaling pathway is affected (51, 52, 57). Therefore, also emerges as key player in regulation of cell metabolism, cell homeostasis, T cell polarization and activation (also in terms of viral infections as discussed below) and new, exciting avenues are opened if taking into account this ´old-fashioned´ component of innate immunity.

## Complement, HIV-1 and DCs

As illustrated above, both DCs and complement comprise a bridge between innate and adaptive immunity—but which outcome does a combined action of complement and DCs exert on HIV-1 infection and antiviral activity?

DCs abundantly express CR3 and CR4—both are members of the beta-2 (CD18) integrins and in particular CR4, comprised of CD11c and CD18, is a specific marker for this cell type (58). CR3 and CR4 are involved in binding multiple ligands, participating in phagocytosis, immune synapse formation, leukocyte trafficking, and co-stimulation. Although both receptors are thought to react in a similar way because of their homology, differences with respect to their structural cellular immunology were recently reviewed by Vorup-Jensen and Jensen (2018) (59). From their structure in the metal-ion dependent adhesion site (MIDAS), CR3 mainly binds positively charged species, while in contrast CR4 strongly binds negative-charged species—therefore, also different functions with respect to CR3 and CR4 are obvious, yet neglected. Both, CR3 and CR4 with their multitude of ligands, were considered as scavenger receptors that facilitate removal of decayed material (60, 61). As such, inflammation by several types of leukocytes is down-modulated by outside-in signaling (62)—this attitude fits well in case of CR3, known to clear opsonized apoptotic cells, immune aggregates or other species present in plasma without triggering a pro-inflammatory response (63). Defects in CR3 or complement component deficiency were shown to be associated with autoimmune diseases such as systemic lupus erythematosus due to failures in cellular clearance (63). Therefore, CR4 was also put into the convenient drawer of being a scavenger receptor—but as suggested by Vorup-Jensen et al., due to its binding to polyanionic species, CR4 may serve quite different functions and act as an immune receptor to alert the immune system to microbial hazards (59). We also highlighted this versatility of CR3 and CR4 on DCs with respect to anti-HIV-1 immunity very recently (64). Among the first cells encountering HIV-1 are CR3/CR4-expressing DC subsets, such as mucosal Langerhans Cells (LCs) or dermal DCs and CR3-expressing macrophages and monocytes. Since HIV-1 spontaneously activates the classical complement pathway even in seminal fluid (2) and is very well protected against complement-mediated lysis due to acquiring RCAs during the budding process (65), interactions with these CR-expressing cells will be much more likely compared to the well described ligation of C-type lectin receptors (CLRs) at mucosal surfaces. While non-opsonized HIV-1 poorly replicates in DCs, covalently linked iC3b fragments on the HIV-1 surface were illustrated to significantly enhance *de novo (cis)* infection of CR3/CR4-expressing DC subsets (LCs, dermal DCs, blood BDCA1^+^ DCs) and CR3-expressing monocytes and macrophages (7–10, 46, 66–71). CR3 is directly interacting with the HIV-1 envelope gp41 due to four homologous C3 regions (49, 72). Several studies have shown that semen affects the efficiency of HIV-1 transmission (2, 67, 73), and we found enhanced LC infection *in vitro* and *ex vivo* due to pre-treatment of HIV-1 with semen (unpublished). While semen was illustrated to contain components like mucin 6, that prevent transmission of HIV-1 to CD4^+^ T cells by blocking DC-SIGN-mediated transfer of HIV-1 from DCs to T cells (74, 75), mucosal LCs were found to efficiently attract LCs to the site of the infection (76, 77) and capture semen-opsonized HIV-1 *via* CR-mediated interactions (unpublished). HIV-1 infection of monocyte-derived DCs as well as dermal and BDCA1^+^ DCs *in vitro* and *ex vivo* was illustrated to work in a C-type lectin independent manner, when opsonized with complement, while interactions with non-opsonized HIV-1 were significantly inhibited upon blocking DC-SIGN interactions (46). The poor replication of non-opsonized HIV-1 in these DCs is mainly mediated *via* the restriction factor SAMHD1 and the non-permissiveness of DCs is suggested to go along with viral evasion (**Figure 2**). SAMHD1 restricts HIV-1 infection in DCs, myeloid cells and resting CD4^+^ T cells by blocking reverse transcription due to limiting the dNTP pool - thus HIV-1 infection is prevented and efficient antiviral DC activation avoided (17, 20–22, 78). Other studies revealed infection of DCs with non-opsonized HIV-1 but lack of activation and type I IFN responses (79, 80). Gringhuis et al. (81) showed that low type I IFN responses in HIV-1-exposed DCs was due to DC-SIGN-dependent activation of Raf-1 and PLK1, that inhibited downstream IRF3 activation (81). When SAMHD1 restriction in DCs was revoked by Vpx of HIV-2 or SIVsm, DCs were infected to higher levels and illustrated higher maturation, co-stimulatory capacity as well as a potent type I IFN induction (13). Potent antigen presentation and initiation of antiviral immunity *via* humoral factors and T cells probably contribute to the slower pathogenesis of HIV-2 or control of SIV in natural hosts. These features could make HIV/SIV visible to the immune system, and all—DC maturation, upregulation of co-stimulatory molecules on DCs and stimulation of antiviral immunity - were also mediated by complement-opsonized HIV-1 and interactions with CRs 3 and/or 4 on DCs (8–10, 64, 68). HIV-C was demonstrated to overcome restriction in monocyte-derived and blood BDCA1^+^ DCs by SAMHD1 phosphorylation at the T592 residue and not due to degrading SAMHD1 similar to Vpx (9, 21). The complement-mediated SAMHD1 phosphorylation was associated with significantly enhanced DC infection, DC activation as well as aberrant type I interferon expression, pro-inflammatory responses and stronger induction of cytotoxic T cell induction by complement-HIV-exposed DCs (8–10, 68). The two major regulators of type I IFN responses comprise IRF3 and IRF7 (82). When DCs were exposed to HIV-C, Posch et al. (9) highlighted that SAMHD1 phosphorylation in DCs was associated with higher STAT1/NFκB activation and IRF3 translocation to the nucleus and the cells exerted a higher T cell stimulatory capacity and higher antiviral potential. Manel et al., also demonstrated that, if restriction to infection was relieved by addition of Vpx, newly synthesized HIV-1 capsids mediated an efficient DC activation and antiviral immune responses *via* IRF3 and CYPA (13). Also Su et al. showed that co-culture with CD4^+^ T cells decreased SAMHD1 levels in DCs, thus enhancing HIV-1 replication accompanied by DC maturation and increased type I IFN secretion from the cells (83). In disagreement to these data, another study demonstrated that despite higher DC infection by complement-opsonized HIV-1 a decreased antiviral immune response was observed from these DCs (84). Despite an enhanced IRF3 activation was observed also in those DCs after stimulation with complement-opsonized HIV-1, a dampening of the immune response with decreased antiviral and inflammatory capacity was mediated in these studies (69, 84). The observed discrepancies might rely on differential expression of CR3 and CR4, either linked to a scavenger (CR3) or immune (CR4) function as suggested by Vorup-Jensen and Jensen (59) and lately shown in terms of HIV-C/DC interactions by Bermejo-Jambrina et al. (64). Very recently we identified CR4 as the receptor responsible for contributing to a DC-driven higher immune response to complement-opsonized HIV-1. In contrast, inflammatory cytokine production was restricted and dampened *via* CR3 (64). Therefore, either a dampening of anti-HIV-1 immune responses may be observed in DCs mainly expressing CR3, while DCs expressing both, CR3 and CR4, at high levels may initiate an inflammatory reaction. This new knowledge might be exploited to target CR3 and CR4 in an antithetic manner to switch off (CR3) or on (CR4) inflammatory and type I IFN responses.

## Complement, HIV-1 and Chronic Immune Activation

Little is known with respect to the role of complement in chronic immune activation. Effects during chronic disease mediated by complement are hard to investigate due to the presence of HIV-1-specific antibodies also binding to the viral surface and interacting with Fcγ receptors on DCs (68). These bound and non-neutralizing HIV-1 antibodies were demonstrated to attenuate the capacity of DCs to stimulate HIV-specific CTLs (68), but their impact on chronic immune activation still needs to be determined. We recently described a significantly enhanced type I IFN as well as pro-inflammatory immune response, when monocyte-derived or blood DCs were exposed to HIV-C (9, 10, 64). Therefore, complement coating of HIV-1 makes the virus visible for DCs, resulting in DC maturation, CTL induction and high immune activation, which is probably of advantage during the acute phase of HIV-1 pathogenesis. As shown by Gringhuis et al. (81) non-opsonized HIV-1 normally escapes immune surveillance by DCs due to blocking the DDX3-MAVS axis *via* CLR signaling. In contrast, when inhibiting DDX3-MAVS blockade an efficient type I interferon response and DC maturation were induced in HIV-1-infected monocyte-derived DCs and in infected individuals (81). Also in HIV-C-exposed DCs these features of improved early type I IFN responses, maturation and CTL-stimulation were monitored (8–10, 64), and in a yet unpublished work, we found that complement opsonization of HIV-1 mediates activation of DCs *via* MAVS. As found in rhesus macaques, blocking type I IFN responses early during SIV infection decreased expression of antiviral genes, increased SIV reservoir size and CD4^+^ T cell depletion (85). However, permanent exposure to type I IFN responses might lead to desensitization and increase in disease progression (85). Therefore, complement is a two-edged sword - during acute HIV-1-infection, where DCs are a prominent target for HIV-1, it improves DC activation, type I IFN induction and thus limits viral replication. But type I IFNs might contribute to disease progression, if continuously activating more T cells and thus generating more targets during HIV-1 infection and thus driving progressive CD4^+^ T cell loss (86, 87). As discussed in the paragraph above targeting either CR3 or CR4 could provide a novel strategy to specifically regulate type I IFN and pro-inflammatory immune responses.

## Conclusions

Covalent complement deposition on HIV-1 particles, observed during all steps of viral pathogenesis and its subsequent interactions with DCs, comprise mixed blessing. In the acute phase of HIV-1 infection, commonly associated with a significant reduction in viral load by efficient early type I IFN induction and effective priming of HIV-specific cytotoxic T lymphocytes, complement coating of HIV-1 particles seems to be beneficial due to making the virus more visible for DCs, the most potent antigen-presenting cells of our immune system. This higher visibility may be associated with more effective DC maturation and activation *via* CR4 resulting in extensive stimulation of anti-HIV-1 immune mechanisms thereby guiding control of viral replication. A dampening of antiviral immunity at the beginning of infection *via* C-type lectins, in case of non-opsonized HIV-1, or CR3 in case of opsonized HIV-1, might subvert DC function at mucosal sites. Viral control by type I interferons may be minimized and proper activation of cytotoxic T cell responses impeded. During chronic phase, triggering the ´wrong´ signal on DCs may be associated with extensive antiviral type I interferon responses and chronic inflammation thus driving HIV-1 pathogenesis. Overall, characterizing the versatile receptors CR3 and CR4 on DCs in the minutest detail might open new avenues of therapeutic interventions of DCs during HIV-1 infection.

## Author Contributions

Writing—original draft, MB-J, WP, DW. Writing—Review and editing, MB-J, WP, CL-F, DW. All authors contributed to the article and approved the submitted version.

## Funding

We thank the Austrian National Bank (OeNB Jubiläumsfonds, number 17614 to WP) and the Austrian Science Fund FWF, project #: P33510-B (to DW).

## Conflict of Interest

The authors declare that the research was conducted in the absence of any commercial or financial relationships that could be construed as a potential conflict of interest.

The handling editor declared a shared affiliation, though no other collaboration with the authors.

## Abbreviations

Ag, Antigen; AP, Alternative complement pathway; APCs, Antigen-presenting cells; C, Complement; CCR, CC- Chemokine receptor; CDK1, Cyclin dependent kinase 1; CDK2, Cyclin dependent kinase 2; cGAS, cyclic GMP-AMP Synthase; CLR(s), C-type lectin Receptor(s); CP, Classical complement pathway; CR(s), Complement receptor(s); CTLs, Cytotoxic T cells; CYPA, Cyclophilin A; CXCR, CXC chemokine receptor; DC(s), Dendritic cell(s); DC-SIGN, Dendritic cell-specific intercellular adhesion molecule-3-grabbing non-integrin; dNTPs, Deoxynucleotide triphosphate; HIV-1, Human immunodeficiency virus type 1; HIV-2, Human immunodeficiency virus type 2; HIV-C, Complement opsonized HIV; ICs, Immune Complexes; iDCs, Immature dendritic cells; IRF, Interferon regulatory factors; LCs, Langerhans cells; LP, Lectin complement pathway; MAC, Membrane attack complex; MAPKs, Mitogen-activated protein kinase; MBL, Mannose-binding lectin; MHC II, Major histocompatibility complex class II; MIDAS, Metal-ion dependent adhesion site; NF-кB, nuclear factor kappa-light-chain-enhancer of activated B cells; PAMPs, Pathogen associated molecular patterns; PLK1, Polo-like kinase 1; PRRs, Pattern Recognition Receptors; RCAs, Regulators of complement activation; SAMHD1, SAM domain- and HD domain- containing protein 1; SIV, Simian immunodeficiency virus; STAT1, Signal transducer and activator of transcription 1; STING, Stimulator of interferon genes; Vpx, Viral protein X.
